# Immune Responses following Stereotactic Body Radiotherapy for Stage I Primary Lung Cancer

**DOI:** 10.1155/2013/731346

**Published:** 2013-11-10

**Authors:** Yoshiyasu Maehata, Hiroshi Onishi, Kengo Kuriyama, Shinichi Aoki, Masayuki Araya, Ryo Saito, Licht Tominaga, Mitsuhiko Oguri, Tsutomu Araki

**Affiliations:** ^1^Department of Radiation Oncology, Kofu Municipal Hospital, 366 Masutsubo, Kofu, Yamanashi 4000832, Japan; ^2^Department of Radiation Oncology and Radiology, University of Yamanashi, School of Medicine, 1110 Shimokato, Chu-o, Yamanashi 4093898, Japan

## Abstract

*Purpose*. Immune responses following stereotactic body radiotherapy (SBRT) for stage I non-small cell lung cancer (NSCLC) were examined from the point of view of lymphocyte subset counts and natural killer cell activity (NKA). *Patients and Methods*. Peripheral blood samples were collected from 62 patients at 4 time points between pretreatment and 4 weeks post-treatment for analysis of the change of total lymphocyte counts (TLC) and lymphocyte subset counts of CD3^+^, CD4^+^, CD8^+^, CD19^+^, CD56^+^, and NKA. In addition, the changes of lymphocyte subset counts were compared between patients with or without relapse. Further, the correlations between SBRT-related parameters and immune response were analyzed for the purpose of revealing the mechanisms of the immune response. *Results*. All lymphocyte subset counts and NKA at post-treatment and 1 week post-treatment were significantly lower than pre-treatment (*P* < 0.01). No significant differences in the changes of lymphocyte subset counts were observed among patients with or without relapse. The volume of the vertebral body receiving radiation doses of 3 Gy or more (VV_3_) significantly correlated with the changes of nearly all lymphocyte subset counts. *Conclusions*. SBRT for stage I NSCLC induced significant immune suppression, and the decrease of lymphocyte subset counts may be associated with exposure of the vertebral bone marrow.

## 1. Introduction

Surgical resection is the standard treatment for stage I primary non-small cell lung cancer (NSCLC) [1]. However, a proportion of stage I NSCLC patients are considered medically inoperable because of comorbidities and advanced age. Moreover, certain patients who meet the criteria for surgery refuse such intervention for various reasons. In contrast, stereotactic body radiotherapy (SBRT) is known to be an especially valuable therapeutic option in such patients [2, 3]. SBRT is a radiation technique that provides precisely targeted high-dose irradiation to a tumor while minimizing radiation delivery to adjacent normal tissues. This targeting allows treatment of small-sized tumors in a limited number of single-high dose fractions. Surgical resection is invasive and causes stress reactions. Those stress reactions are known to lead immune suppression, some of which appear as a postoperative decrease in lymphocyte counts and NK cell activity (NKA). Among lymphocytes, the CD3^+^, CD4^+^, CD8^+^, CD19^+^, and CD56^+^ subsets are considered to be important in antitumor immunity. Expression of CD3^+^ and CD4^+^ correlates with overall survival in NSCLC patients [4]. CD8^+^ and CD56^+^ cells exert antitumor activity via antigen specific and antigen nonspecific mechanisms [5, 6]. Further, elevated circulating CD19^+^ lymphocytes can predict survival in patients with gastric cancer [7]. NKA is also considered to be important in the immune suppression caused by surgical resection and is known as a good indicator of the host's resistance against the tumor [8–11]. In addition, the extent and duration of this immune suppression are affected by the magnitude of surgical invasiveness [12–14]. Furthermore, this immune suppression may increase the risk of tumor growth and metastasis [15–20]. In comparison, relatively little is known regarding the effect of SBRT on immune function in patients with lung cancer. Thus, the purpose of this study was to evaluate the effect of SBRT on immune function by examinations as follows: changes of peripheral lymphocyte counts and NKA after SBRT; mechanisms of those changes; relationship between those changes and cancer relapse.

## 2. Patients and Methods

### 2.1. Patients and Treatments

Thirty-six patients with stage I NSCLC who underwent treatment with SBRT from August 2008 to March 2010 were studied (group I), and changes of lymphocyte subset counts were examined at pretreatment and post-treatment. Then, patients of group I were divided into a relapse group (IR+) and nonrelapse group (IR−) according to being with or without relapse within 2 years after SBRT, and degree of changes in lymphocyte subset counts was compared between both groups. As a significant decrease of lymphocyte subsets counts at post-treatment in group I was confirmed, 26 patients with stage I NSCLC who underwent treatment with SBRT from April 2010 to March 2012 were studied (group II). For the purpose of examining the immune response induced by SBRT in more detail, NKA as well as lymphocyte subset counts was examined at 4 time points, pretreatment, post-treatment, 1 week post-treatment and 4 weeks post-treatment in group II. Patient characteristics were shown in Table 1. SBRT was performed by three-dimensional treatment plans using 10 different noncoplanar dynamic arcs with a linear accelerator (EXL-15DP, Mitsubishi Electric, Tokyo, Japan). A total dose of 40–70 Gy was delivered in 4 to 10 fractions at the minimum dose point in the planning target volume (PTV) using a 6 MV X-ray. Patients who underwent chemotherapy within 4 weeks before SBRT were excluded, and no patients were treated with chemotherapy or steroids during SBRT or by 4 weeks after completion of SBRT.

### 2.2. Blood Sampling and Analysis

Peripheral blood samples were collected 1–3 days before initiation of SBRT (pretreatment) and just after completion of SBRT (post-treatment) in group I. The samples in group II were collected at 4 time points: pretreatment, post-treatment, 1 week post-treatment and 4 weeks post-treatment as described above. All blood samples were drawn at 9:00-10:00 a.m. after overnight fasting to avoid diurnal lymphocyte changes. All peripheral blood samples were stained for flow cytometric analysis using CYTO-STAT tetraCHROME monoclonal antibody, followed by analysis of total lymphocyte counts (TLC) and lymphocyte subset counts of CD3^+^, CD4^+^, CD8^+^, CD19^+^, and CD56^+^ with a Cytomics FC500 Flow Cytometry System (Beckman Coulter, Inc., Miami, FL, USA) with a 488 NM argon laser. NKA was assessed by chromium-51 release assay to measure radioactivity released from ^51^Cr-loaded K562 target cells (SRL, Inc., Tokyo, Japan).

### 2.3. SBRT-Related Parameters

In group II, SBRT-related parameters were calculated using XiO treatment planning system (CMS Inc., St. Louis, MO, USA), and correlations between those parameters and degree of immune suppression were analyzed for the purpose of revealing the mechanisms of the immune suppression. The SBRT-related parameters included planning target volume (PTV), mean total dose to the PTV (MTD_PTV_), and volume of the vertebral body (VV) receiving radiation doses of 3, 5, 10 Gy, or more (VV_3_, VV_5_, and VV_10_, resp.). PTV was the real irradiated target volume and was uniformly created by expanding the gross tumor volume by 2–5 mm as an internal margin mainly according to the inaccuracy of breath holding and by 5–10 mm as setup margins in the 3 dimensions. VV_3_, VV_5_, and VV_10_ were regarded to imply a degree of influence for vertebral bone marrow by radiation.

### 2.4. Statistical Analysis

Differences in changes of lymphocyte subset counts and NKA were analyzed by the paired *t*-test. Differences of changes of lymphocyte subsets counts between group IR+ and IR− were examined by the Mann-Whitney *U* test. Correlations between SBRT-related parameters and the changes of lymphocyte subset counts and NKA were analyzed with linear regression. In all tests, the difference was considered significant when the  *P*  value was less than 0.05.

### 2.5. Ethical Approval and Informed Consents

This study was approved by the Ethical Committee of the University of Yamanashi, School of Medicine, and conducted according to the Helsinki Declaration of 1975, as revised in 2000. Patients were interviewed prior to acceptance in the study and were given written and verbal information regarding the study. All patients provided written, informed consent.

## 3. Results

### 3.1. Group I

**Figure 1:**
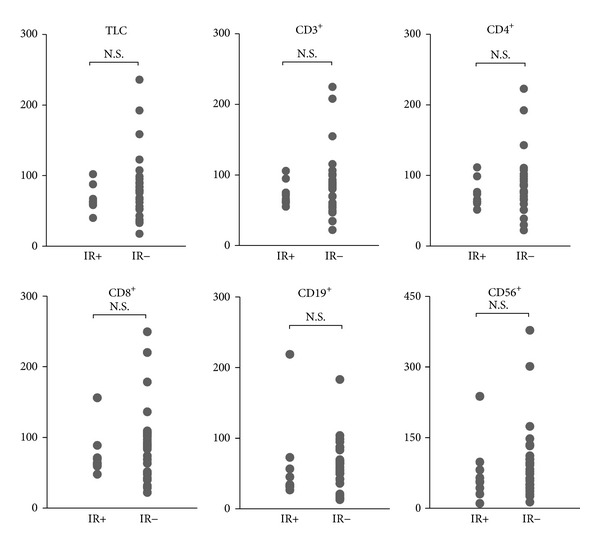
Comparison of the ratio of each lymphocyte subset count post-treatment between groups IR+ and IR−, assuming values at pretreatment as 100. No significant differences were observed in each lymphocyte subset using Mann-Whitney *U* test.

All patients completed the treatment. The changes of lymphocyte subsets counts at pretreatment and post-treatment in the group I were shown in Table 2. All lymphocyte subset counts at post-treatment were significantly lower than at pretreatment (*P* < 0.01). In group I, all patients were followedup over 2 years after completion of treatment, and 9 patients (27.3%) experienced relapse, including local recurrence, lymph node metastases, and distant metastases (group IR+). No significant differences were observed in post-treatment to pretreatment ratio of each lymphocyte subset count between groups IR+ and IR− using Mann-Whitney *U* test (Figure 1).

### 3.2. Group II

**Figure 2:**
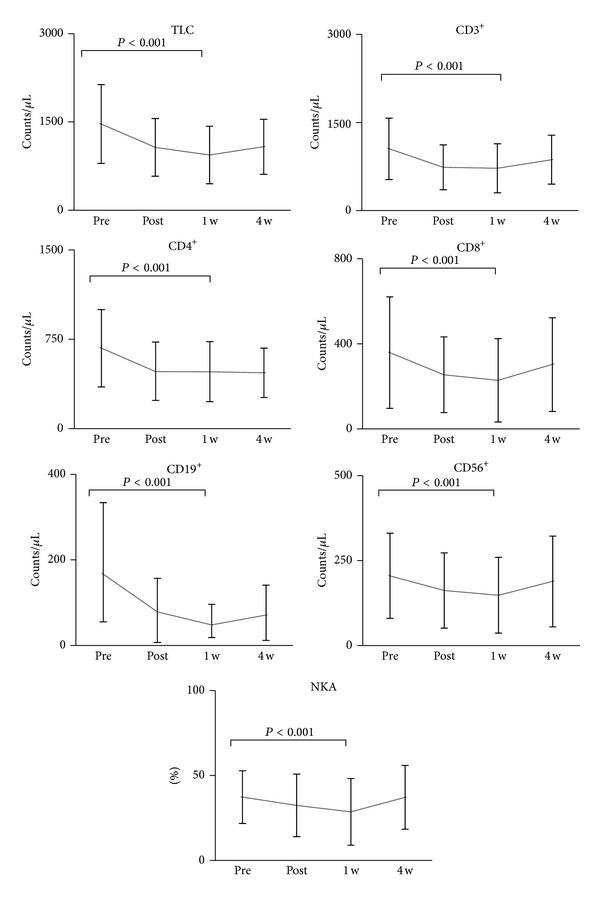
Mean value of lymphocyte subsets counts with standard deviations for 4 time points in group II. All subsets at 1 week post-treatment were significantly lower than at pretreatment. TLC, CD4^+^, and CD19^+^ at 4 weeks post-treatment were still lower than those at pretreatment, though nearly all subsets counts at 4 weeks post-treatment were higher than at 1 week post-treatment. Pre: pretreatment; Post: post-treatment; 1 w: 1 week post-treatment; 4 w: 4 weeks post-treatment.

**Figure 3:**
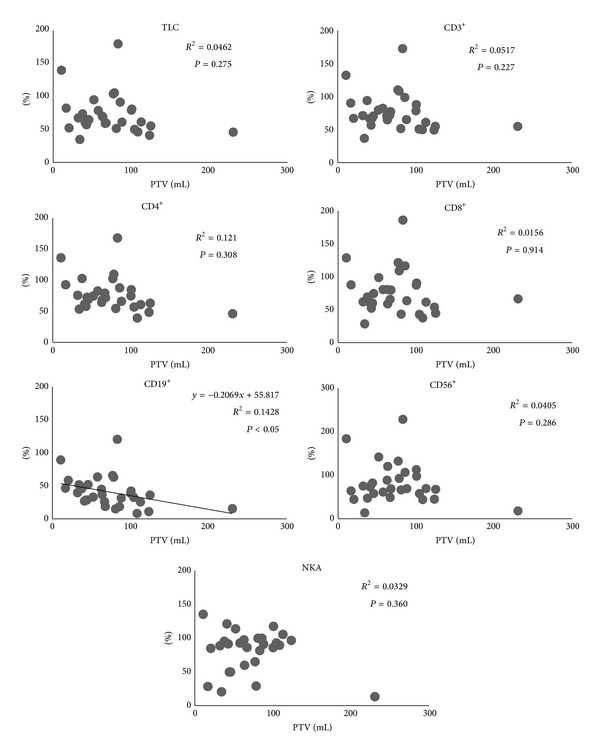
Correlations between planning target volume (PTV) and the ratio of each lymphocyte subset count and NKA at 1 week post-treatment, assuming values at pretreatment as 100. PTV correlated with none of the lymphocyte subsets except CD19^+^.

**Figure 4:**
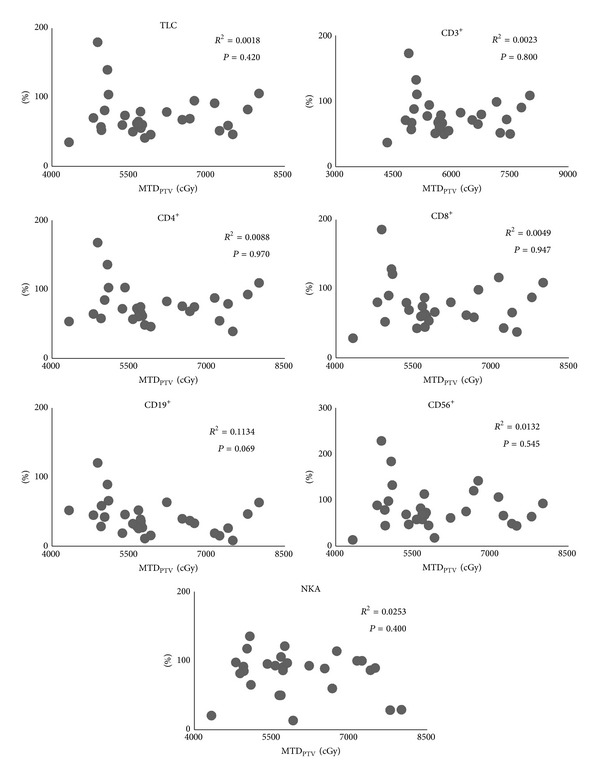
Correlations between mean total dose of PTV (MTD_PTV_) and the ratio of each lymphocyte subset count and NKA at 1 week post-treatment, assuming values at pretreatment as 100. No correlations were observed for any lymphocyte subsets or NKA.

**Figure 5:**
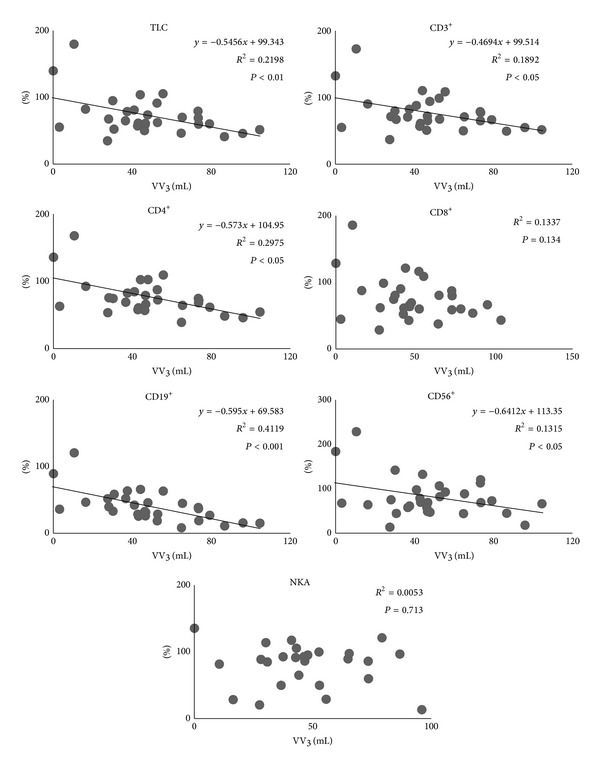
Correlations between the volume of the vertebral body receiving radiation doses of 3 Gy or more (VV_3_) and the ratio of each lymphocyte subset count and NKA at 1 week post-treatment, assuming values at pretreatment as 100. VV_3_ significantly correlated with the ratios of TLC, CD3^+^, CD4^+^, CD19^+^, and CD56^+^.

**Figure 6:**
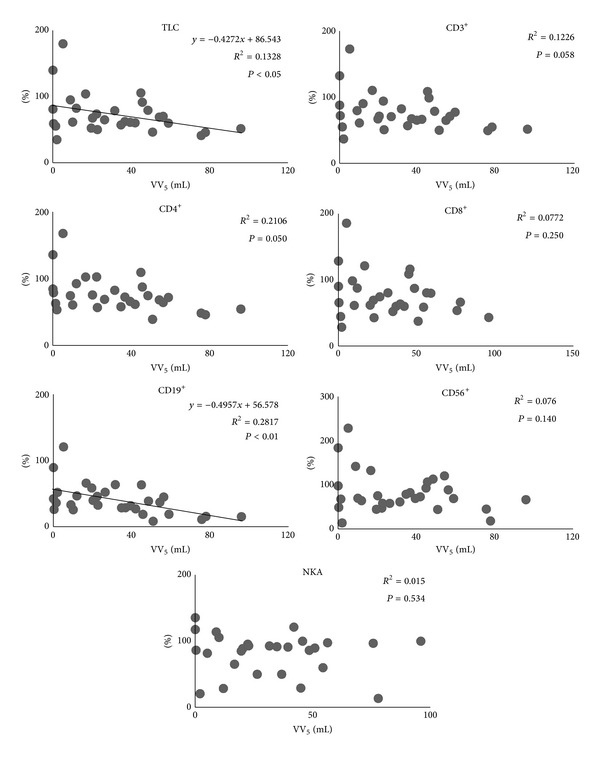
Correlations between the volume of the vertebral body receiving radiation doses of 5 Gy or more (VV_5_) and the ratio of each lymphocyte subset count and NKA at 1 week post-treatment, assuming values at pretreatment as 100. VV_5_ significantly correlated with the ratios of TLC and CD19^+^.

**Figure 7:**
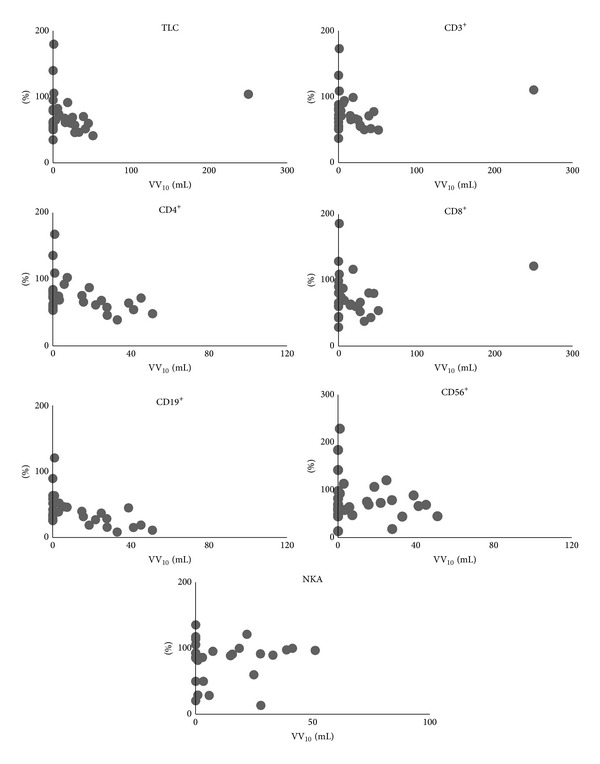
Correlations between the volume of the vertebral body receiving radiation doses of 10 Gy or more (VV_10_) and the ratio of each lymphocyte subset and NKA at 1 week post-treatment, assuming values at pretreatment as 100. No correlations were observed for any lymphocyte subsets or NKA.

All patients completed the treatment. All lymphocyte subset counts and NKA at 1 week post-treatment were significantly lower than at pretreatment (*P* < 0.01). Besides, TLC, CD4^+^, and CD19^+^ at 4 weeks post-treatment were significantly lower than at pretreatment, though nearly all lymphocyte subsets counts at 4 weeks post-treatment were higher than at 1 week post-treatment (Figure 2). The relationship between SBRT-related parameters and the ratio of each lymphocyte subsets count and NKA at 1 week post-treatment, assuming values at pretreatment as 100, was as follows. PTV correlated with none of the lymphocyte subsets except CD19^+^ (Figure 3). MTD_PTV_ correlated with none of the lymphocyte subsets (Figure 4). VV_3_ correlated significantly with all lymphocyte subsets except CD8^+^ (Figure 5). VV_5_ correlated significantly with TLC and CD19^+^ (Figure 6), and VV_10_ correlated with none of the lymphocyte subsets (Figure 7).

## 4. Discussion

Immune responses induced by conventional radiotherapy for lung cancer have been described previously, and it is known that peripheral lymphocyte subset counts are reduced after radiotherapy [21–23]. However, those studies dealt with mostly stage III or IV lung cancer patients undergoing radiotherapy with large irradiation fields. In contrast, the present study is the world's first study showing that SBRT for stage I NSCLC resulted in suppression of lymphocyte subset counts and NKA. Furthermore, the study showed not only the decrease in lymphocyte subset counts and NKA but also persisting of the decrease for 1 to 4 weeks after completion of SBRT. Interleukin 2 (IL-2) produced by lymphocytes is well known to be related to cell-mediated immunity. Persistent lymphopenia could be considered to reduce synthesis of IL-2 and cause immune suppression.

Immune suppression in cancer patients is associated with recurrence or metastasis. The present study explored the hypothesis that acute decrease of lymphocyte subset counts induced by SBRT for single thoracic malignant lesions was associated with relapse after SBRT. However, the results demonstrated no significant differences between the recurrence group and nonrecurrence group on the change of each lymphocyte subset count at post-treatment. Further study in a larger number of patients, according to the specific type of relapse, and focusing on the time-dependent changes of each lymphocyte subset count, would be of benefit.

Several mechanisms by which SBRT induced a decrease in peripheral lymphocytes could be considered. Firstly, it is possible that circulating lymphocytes in peripheral vessels were directly killed by the radiation-associated photons. In this situation, the decrease in lymphocyte count would be proportional to the irradiated volume. Dallüge et al. reported that the lymphocyte count decreased to a larger degree with pendulum therapy than with stationary field radiation in patients undergoing mediastinal radiotherapy, suggesting that the decrease in the lymphocyte count was due to irradiation of lymphocytes in the blood stream rather than the irradiation of the bone marrow [24]. However, in the present study, the decrease in lymphocyte subsets (with the exception of CD19^+^) did not correlate with PTV, suggesting that direct damage was not the mechanism by which SBRT induced a decrease in the lymphocyte count. Surgical resection can cause a stress reaction and immunosuppression via activation of the hypothalamic-pituitary-adrenal axis [25, 26]. Furthermore, the extent and duration of the suppression are related to the magnitude of surgical invasiveness [13, 14]. Thus, a stress reaction induced by SBRT could conceivably result in a decrease in the peripheral lymphocyte count. In that situation, the decrease in lymphocyte count should be proportional to the total radiation dose. However, in the present study, no correlation was seen between MTD_PTV_ and lymphocyte count, suggesting that the SBRT-induced decrease in lymphocyte count was unlikely to be mediated by a stress reaction. Another possible mechanism for SBRT-induced decrease in lymphocyte count is radiation-induced myelosuppression. This was supported by observations from the present study in which VV_3_ significantly correlated with nearly all lymphocyte subsets, and VV_5_ correlated with TLC and CD19^+^. Though VV_10_ correlated with none of lymphocyte subsets, it was likely to result from radiotherapy planning with the goal of minimizing radiation delivery to the vertebral body.

NKA was also reduced at 1 week after completion of SBRT. However, in contrast to the lymphocyte subsets counts, NKA at 1 week post-treatment did not correlate with the volume of the vertebral body. This fact suggests that the decrease in NKA is not related to radiation delivery to the vertebral body. Rather, this phenomenon may be related to an SBRT-induced stress reaction. 

This study has several limitations. First, it employed relatively few time points for blood sampling. This study showed that TLC, all lymphocyte subsets, and NKA continued to decrease for at least 1 week and improved slightly at 4 weeks after treatment without reaching pretreatment levels. However, the temporal pattern of changes during this time is not precisely clear, as blood sampling was not performed between 1 week after treatment and 4 weeks after treatment. Conducting blood sampling of additional time points may help clarify the temporal course of this phenomenon. Second, this study did not utilize a control group; comparison against patients who did not receive SBRT to those undergoing conventional radiotherapy or surgical resection within a prospective study would be of benefit.

In summary, SBRT for stage I NSCLC induced significant immune suppression that manifested by a decrease in lymphocyte counts and depression of NKA. The decrease in lymphocyte subset counts may be due to a lymphocyte production disorder caused by exposure of the vertebral bone marrow to radiation, while the depression of NKA may be related to a stress reaction caused by SBRT. Though SBRT which is considered as a low-invasive treatment is performed especially for patients with severe comorbidities and advanced age, this study showed that there is room for improvement in the point of view of immune suppression. In the future, we will advance research for the purpose of analyzing relations between immunodynamics and prognosis with larger patient numbers, more frequent blood sampling, comparison with appropriate control or experimental groups, and comparison of fractionations and radiation techniques.

## Figures and Tables

**Table 1:** Patient characteristics.

	Group I	Group II
	(*n* = 36)	(*n* = 26)
Sex		
Male	25	17
Female	11	9
Age (years)	61–90	64–89
	(median: 80)	(median: 81)
Dose/fractionation		
40–60 Gy/4 fr	29	20
60–70 Gy/10 fr	7	6

**Table 2:** Mean lymphocyte subsets counts at pretreatment and post-treatment in group I.

	Pre (counts/*µ*L)		Post (counts/*µ*L)		*P* value
	Av	S.D.	Av	S.D.	(paired *t*-test)
TLC	1363	658	917	352	<0.001
CD3^+^	872	480	636	277	<0.001
CD4^+^	566	287	424	194	<0.001
CD8^+^	299	252	209	132	<0.01
CD19^+^	162	116	80	65	<0.001
CD56^+^	306	254	188	108	<0.01

Pre: pretreatment; Post: post-treatment; Av: average values; S.D.: standard deviations; TLC: total lymphocyte counts.
